# 29例肺粘液表皮样癌的临床分析

**DOI:** 10.3779/j.issn.1009-3419.2017.03.05

**Published:** 2017-03-20

**Authors:** 晶晶 侯, 慧娟 王, 国伟 张, 晏阳 黄, 智勇 马

**Affiliations:** 450008 郑州，郑州大学附属肿瘤医院呼吸内科 Department of Respiratory Medicine, the Affiliated Cancer Hospital of Zhengzhou University, Zhengzhou 450008, China

**Keywords:** 肺肿瘤, 粘液表皮样癌, 临床特征, 治疗, 预后, Lung neoplasms, Mucoepidermoid carcinoma, Clinical features, Treatment, Prognosis

## Abstract

**背景与目的:**

肺粘液表皮样癌（mucoepidermoid carcinoma, MEC）是罕见的肺部恶性肿瘤，其临床特征及预后尚不完全明确，本研究旨在探讨肺粘液表皮样癌的临床特征、诊治方法及预后影响因素。

**方法:**

回顾郑州大学附属肿瘤医院2006年1月-2015年12月收治的29例肺MEC患者的临床资料，对临床特征、诊断方法、治疗措施及预后进行分析。

**结果:**

全组29例患者占同期新诊断肺癌的0.16%（29/18, 021），其中男性18例，女性11例；中位年龄45岁（10岁-79岁）；高级别肺MEC 17例，低级别12例。6例患者行表皮生长因子受体（epidermal growth factor receptor, *EGFR*）基因检测均未检测到突变。行手术为主的综合治疗17例，非手术治疗12例。中位随访时间35（5-114）个月，至随访结束，17例患者已死亡。全组1年、3年、5年生存率分别为65.5%、51.2%和39.4%，中位生存时间37个月。

**结论:**

肺MEC发病率低，临床表现缺乏特异性，确诊主要依据术后组织病理，并辅以免疫组化标记结果，手术是主要的治疗方法，病理级别以高级别为主，其预后与组织学分级、临床分期密切相关，EGFR酪氨酸激酶抑制剂（EGFR-tyrosine kinase inhibitor, EGFR-TKI）有望改善肺MEC的预后。

## Abstract (en)

**Background and objective:**

Pulmonary mucoepidermoid carcinoma (MEC) is an extremely rare pulmonary malignant tumor, its clinical features and conditions of prognosis is not entirely clear. The aim of this study is to discuss clinical features, diagnostic and therapeutic methods, and prognosis of pulmonary MEC.

**Methods:**

We retrospectively studied 29 pulmonary MEC patients who diagnosed from January 2006 to December 2015 in Affiliated Hospital of Zhengzhou University. The clinical features, prognosis, diagnostic and therapeutic methods were analyzed.

**Results:**

There were 20 patients identified as pulmonary MEC, which constitutes 0.18% of all the lung tumor patients. There were 18 males and 11 females, the median age of the patients was 45 years (range 10-79). There were 17 patients identified as high-grade pulmonary MEC and 12 low-grade. Epidermal growth factor receptor (*EGFR*) mutation detection was performed in six patients, none was positive. 17 cases was underwent surgery based comprehensive treatment, 12 cases non-operatived treatment. The median follow-up time was 35 (5-114) months in this cohort of 29 patients. During the follow up, incidence of death was found in 17 cases. The overall 1-, 3-, 5-year survival rates were 65.5%, 51.2%, 39.4%, respectively. The median survival time was 37 months.

**Conclusion:**

The incidence of pulmonary MEC is low, lacking specific clinical characterization. The diagnosis mainly depends on postoperative pathology, aided by immunohistochemical. Surgery is the main treatment method. The majority of pathology was high-grade type. The prognosis of pulmonary MEC closely relates to the pathological types and clinical stage. EGFR-tyrosine kinase inhibitor (EGFR-TKI) is expected to improve the prognosis of pulmonary MEC.

粘液表皮样癌（mucoepidermoid carcinoma, MEC）是由不同比例的表皮样细胞、粘液细胞和中间细胞组成的恶性肿瘤，好发于涎腺，尤其是腮腺，原发于肺部的较为少见，约占肺原发恶性肿瘤的0.1%-0.58%^[1-4]^，因为其发病率较低，相关文献报道也较为少见。本研究回顾29例肺MEC患者的临床资料，分析其临床特征及影响预后的因素，以期提高对本病的认识。

## 1 资料与方法

收集郑州大学附属肿瘤医院2006年1月-2015年12月期间经病理确诊的肺MEC患者共29例，回顾性分析其临床病理特征及影响预后的因素。病理诊断依据2004年世界卫生组织（World Health Organization, WHO）肺、胸膜、胸腺及心脏肿瘤病理学和遗传学分类，定义粘液表皮样癌为出现表皮样细胞、产生粘液的细胞和中间型细胞为特点的恶性上皮肿瘤，组织表现与涎腺同样名称的肿瘤相同。粘液表皮样癌分为高级别和低级别，高级别粘液表皮样癌与腺鳞癌区分较为困难。诊断高级别粘液表皮样癌更典型的标准包括：①外生性支气管内生长；②表面上皮缺乏原位癌改变；③缺乏单个细胞角化和角化珠形成；④有向低级别粘液表皮样癌的移行区。

采用电话或门诊随访，随访截止至2016年4月30日。生存时间自病理确诊开始算起。生存分析采用*Kaplan*-*Meier*法，生存曲线的比较采用*Log*-*rank*检验，将在单因素分析中有统计学意义的变量作为二分类变量，采用*Cox*风险比例模型进行多因素分析。应用SPSS 17.0统计软件进行统计学分析，以*P*＜0.05为差异有统计学意义。

## 2 结果

### 2.1 临床特征

2006年1月-2015年12月我院收治肺MEC 29例，占同期收治肺癌的0.16%（29/18, 021），所有患者均无肿瘤家族史。患者的临床特征详见表 1。患者以男性常见，男女比例为1.6:1，中位年龄45（10-79）岁，吸烟者少见，肿瘤多位于右肺，中央型多见。首发症状无特异性，以咳嗽、咳痰最为常见，病史最短1周最长1年，平均病程为2.5个月。

**1:** 29例肺MEC患者的临床特征 The clinical characteristics of 29 patients with pulmonary MEC

Characteristics	No. of patients
Gender (Male/Female)	
Male	18
Female	11
Median age (yr)	45
Smoking history	
Yes	8
No	21
Range	
Central	25
Peripheral	4
Initial symptoms	
Cough and expectoration	11
Hemoptysis	6
Chest stuffiness	5
Chest pain	3
Cervical lump	2
Headaches	1
Asymptomatic	1
Tumor location	
Right main bronchus	1
Right upper lobe	6
Right middle lobe	4
Right inferior lobe	7
Left upper lobe	7
Left inferior lobe	4
MEC: mucoepidermoid carcinoma; TNM: tumor-node-metastasis.	

### 2.2 胸部影像学表现

15例呈边缘光滑，边界清晰的类圆形结节，6例有浅分叶；14例呈不规则肿块，边界不清；6例出现阻塞性肺炎或肺不张。增强扫描12例肿块均有强化，其中均匀强化1例，不均匀强化11例；轻度强化2例，明显强化10例。肿瘤最大直径为1.1 cm-8.0 cm，平均4.8 cm。

### 2.3 诊断

16例经手术后标本确诊，6例经肺穿刺活检确诊，2例经淋巴结穿刺活检确诊，5例经气管镜确诊。值得注意的是术后确诊的患者中有7例术前曾行气管镜，3例误诊为腺癌，1例误诊为鳞癌，1例误诊为低分化癌，2例未取到组织。依据2004年WHO肺、胸膜、胸腺及心脏肿瘤病理学和遗传学分类，最终17例诊断为高级别肺MEC，12例诊断为低级别肺MEC（HE染色图片见图 1）。6例术后标本和1例肺穿刺标本进行了免疫组化检测（结果见表 2及图 1），其中2例术后标本为气管镜误诊患者。共6例患者的标本进行了表皮生长因子受体（epidermal growth factor receptor, *EGFR*）基因突变检测，均未见*EGFR*基因突变。

**2:** 7例肺MEC的免疫组织化学检测结果 Immunohistochemical staining result of 7 cases of pulmonary MEC

Patient	CK7	P63	TTF-1	Ki67	CK5/6	CK	AB/PAS	Napsin A	SyN
1	+		-	＜10%	-				
2		A few cells+	-	+20%	-		+		-
3	+	+		＜10%	Interspersed +		+		
4	+	+	-	＜10%	+	+			
5	+	+	-	20%	-	+		-	
6	+	+	-	＜10%			+	-	
7	+		-			+			-
Blank: no immunohistochemical staining was performed in this patient.									

**1:**
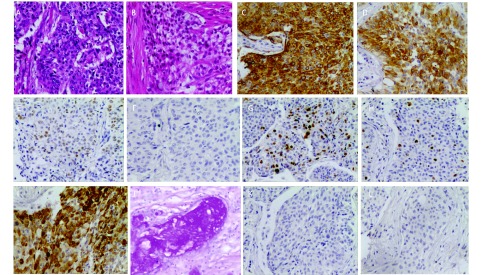
病理与免疫组化检查（×200）。A：高级别肺MEC；B：低级别肺MEC；C：CK；D：CK7；E：P63；F：TTF-1；G: Ki-67；H：Ki-67；I：CK5/6；J：特殊染色（AB/PAS染色）；K：Napsin A；L：SyN。 Pathology and immunohistochemistry (×200). A: high-grade pulmonary MEC; B: low-grade pulmonary MEC; C: CK; D: CK7; E: P63; F: TTF-1; G: Ki-67; H: Ki-67; I: CK5/6; J: special staining (AB/PAS staining); K: Napsin A; L: SyN.

### 2.4 治疗

17例患者接受了手术治疗，其中肺叶切除术11例，全肺切除术5例，开胸探查术1例。6例Ⅰ期患者术后未进行辅助治疗；1例Ⅲa期儿童患者术前拒绝行化疗经讨论后术前给予吉非替尼治疗，18 d后出现病灶完全缓解，行手术治疗；2例术后行化疗加放疗；8例术后行辅助化疗；1例行辅助放疗。化疗方案包括（吉西他滨+顺铂、多西他赛+顺铂，长春瑞滨+顺铂）。未接受手术治疗的12例患者中5例单纯行化疗；4例行化放疗同时或序贯治疗；1例行厄洛替尼治疗；1例Ⅲb期患者行气管镜下肿物切除后口服厄洛替尼治疗；1例仅行支持治疗。化疗方案包括（培美曲塞+顺铂、紫杉醇+顺铂、吉西他滨+顺铂、多西他赛+顺铂、长春瑞滨+顺铂）。

### 2.5 预后

本组患者中位随访时间35个月（5个月-114个月），无失访患者，随访率100%。全部患者1年、3年、5年生存率分别为65.5%、51.2%和39.4%，中位生存期37个月（生存曲线见图 2A）。由于Ⅱ期患者仅有1例，在行分期对预后的影响时将Ⅰ期、Ⅱ期归为一组。影响5年生存率的单因素分析结果显示（表 3，图 2B-图 2D）高级别、低级别分别为11.8%和80.0%（*P*＜0.001），TNM分期Ⅰ期-Ⅱ期、Ⅲ期、Ⅳ期分别为90.0%、16.7%和0.0%（*P*＜0.001），是否行根治性手术治疗分别为65.6%和7.7%（*P*＜0.001）。性别、年龄、是否吸烟、术后是否行辅助放化疗对生存的影响无统计学差异。多因素分析显示TNM分期和病理级别的高低是影响肺粘液表皮样癌的独立危险因素（*P*＜0.05，表 4）。

**2:**
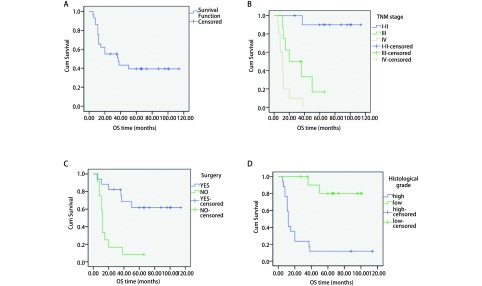
生存曲线图。A：29例患者总体；B：不同TNM分期；C：是否手术；D：不同组织级别。 Survival curves. A: total survival curve of 29 patients; B: according to TNM stage; C: according to surgery; D: according to pathological grade.

**3:** 29例肺MEC的生存的单因素分析 Univariate analysis for the survival of 29 cases with pulmonary MEC

Variables	*n*	Survival rate (%)			Chi-square	*P*
		1-year	3-year	5-year		
Gender					3.607	0.058
Male	18	72.2	44.4	22.2		
Female	11	72.7	63.6	63.6		
Age (yr)					0.706	0.401
≥45	15	73.3	40.0	33.3		
＜45	14	71.4	64.3	45.9		
Smoking history					0.483	0.487
Yes	8	62.5	37.5	37.5		
No	21	81.0	56.7	41.3		
TNM stage					26.891	＜0.001
Ⅰ-Ⅱ	11	100	100	90.0		
Ⅲ	8	75	33.3	16.7		
Ⅳ	10	40	10	0.0		
Radical surgery					16.713	＜0.001
Yes	16	93.8	80.2	65.6		
No	13	53.8	15.4	7.7		
Postoperative adjuvant therapy					1.404	0.236
Yes	9	88.9	66.7	55.6		
No	7	1	1	83.3		
Histologic grade					16.109	＜0.001
High	17	41.2	23.5	11.8		
Low	12	100	90.0	80.0		

**4:** *Cox*回归分析结果 Result of *Cox* regression analysis

Parameters	HR	95%CI	*P*
Histological grade	0.151	0.027-0.844	0.031
TNM stage	7.631	2.057-28.316	0.002
Operative	0.184	0.028-1.220	0.079

## 3 讨论

1952年Smetana等^[5]^首先报道了肺MEC，肺MEC主要起源于大支气管粘膜下层的导管上皮细胞，发病率很低。本组报道的29例肺MEC，占我院同期收治肺部恶性肿瘤患者的0.16%，与文献报道的0.1%-0.58%发病率相似^[1-4]^。肺MEC的发病年龄较轻，中位年龄40岁左右^[6, 7]^，本组最小10岁，最大73岁，中位年龄45岁。性别分布上既往报道并不一致，有文献^[8]^报道多发于男性，也有文献^[2]^报道多发于女性，本组患者男女比例为1.6:1，提示可能更多发于男性。本组仅有8例患者有吸烟史，提示吸烟可能不是肺MEC的主要危险因素，与既往文献^[2, 9, 10]^报道一致。

按粘液细胞、表皮样细胞和中间细胞的构成比例不同将肺MEC分为低级别MEC和高级别MEC^[9]^。低级别MEC以粘液细胞和表皮样细胞常见，中间细胞很少占主导地位，常形成大小不等的囊性结构，高级别MEC以表皮样细胞和中间细胞为主，粘液细胞最少见，常见非典型细胞、核分裂及坏死，肿瘤常以实性成分为主^[11]^。有研究总结5例肺粘液表皮样癌患者的预后认为肺粘液表皮样癌属于低度恶性肿瘤，随后多数文献^[4, 7, 9]^报道以低级别肺MEC为主，但也有文献^[2, 12]^报道以高级别肺MEC为主。本组患者以高级别肺MEC为主，占58.6%。

在临床症状方面肺MEC并无特异性，临床症状主要表现为支气管的刺激和阻塞症状，包括：咳嗽、咳痰、咯血、胸痛、胸闷、发热等，若肿瘤较小或发生于周边也可不引起症状^[12, 13]^。本组25例患者以上述常见症状就诊，3例以转移症状就诊，1例体检时发现。肺MEC计算机断层扫描（computed tomography, CT）影像多表现为肺门或近肺门处边缘清晰的类圆形结节或肿块，部分有浅分叶；肿瘤密度接近或等同于肌肉密度；部分病变内部可出现钙化灶；多伴有阻塞性肺炎、肺不张；增强扫描多表现为明显强化，少数为轻度强化^[14]^。本组病变的CT表现均未见钙化灶，有14例肿块呈不规则形状，与周围边界不清，仅6例出现阻塞性肺炎肺不张，与文献报道有差异。江森等^[14]^报道肿瘤内部出现钙化灶可能与粘液细胞分泌的粘液不完全吸收导致钙盐沉积有关，而低级别肺MEC富含更多的粘液细胞，因此钙化可能更多见于低级别肺MEC，低级别肺MEC由于生长缓慢，多出现气管阻塞性改变，而本组患者多为高级别肺MEC，这可能是本组与文献报道存在影像学差异的原因。

由于肺MEC的发病率低，临床表现和影像学表现缺乏特异性，诊断主要依据组织病理形态特点，而肺MEC的组织学成份复杂，因此，小标本诊断肺MEC存在困难。本组7例术前行气管镜检查未能确诊的患者，最终经术后组织标本确诊。对于小标本及形态学不易确诊的肺MEC患者可以借助免疫组织化学结果辅助诊断。Huo等^[15]^分析了26例肺MEC的免疫组织化学结果发现26例患者CK7、Muc5AC、P63和P40均呈阳性表达，TTF-1均呈阴性表达，Ki-67的变化范围自2%-80%，平均为9.7%，其中高级别肺MEC Ki-67平均为22.4%，低级别肺MEC Ki-67平均为4.1%。Roden等^[16]^报道25例患者中均有P63的表达而无TTF-1和Napsin A的表达，23例有P40的表达。本组7例行免疫组织化学检测，TTF-1、Napsin A检测结果均为阴性，P63、CK7的检测结果均为阳性，4例Ki67＜10%均为低级别肺MEC，2例Ki67 20%均为高级别肺MEC，提示Ki-67指数可作为肺MEC高级别和低级别的辅助鉴别指标。AB/PAS染色阳性为粘液的特异性指标，本组3例患者进行了AB/PAS染色均为阳性，因此推测TTF-1、CK7、Napsin A、P63、Ki67、AB/PAS染色的联合检测或可成为肺MEC的辅助诊断指标。

目前对于肺MEC的治疗仍无统一的标准。但国内外文献对于肺MEC的治疗原则是一致的，均认为可行手术治疗的患者手术完整切除并纵隔淋巴结清扫对于肺MEC的长期生存至关重要^[6, 12, 17]^，晚期不能手术治疗的患者治疗方案同其他非小细胞肺癌。新辅助化疗可用于术前肿块较大的患者，尤其是T4患者^[1]^。术后辅助化放疗的地位仍不确定^[7]^，多数文献认为低级别肺MEC不必行术后辅助放疗或化疗，术后区域淋巴结阳性或肿瘤边缘阳性的推荐行常规放疗或化疗^[1, 7, 12]^。本组16例患者行根治性手术治疗，其中9例术后行辅助治疗，单因素生存分析显示术后是否行辅助治疗生存并无统计学差异。

近年来EGFR酪氨酸激酶抑制剂（EGFR-tyrosine kinase inhibitor, EGFR-TKI）成为存在*EGFR*基因突变肺癌患者的主要治疗手段之一。*EGFR*基因突变多见于肺腺癌，肺MEC的EGFR基因突变结果目前研究尚不多。我国Yu等^[18]^报道了5例（5/20）肺MEC患者存在*EGFR*突变，且突变位点均位于21外显子的L861Q。Xi等^[19]^研究了19例肺MEC的EGFR基因突变状态，结果显示均无*EGFR*基因突变，与本研究结果一致。考虑肺MEC可能存在*EGFR*突变，但不同患者间存在差异，应进一步扩大样本量进行研究。然而，研究发现，即使没有*EGFR*基因突变，仍有许多患者口服EGFR-TKI治疗后获益^[20]^。本组3例患者口服EGFR-TKI治疗，且均无*EGFR*突变，其中1例Ⅲa期患者，口服吉非替尼治疗后出现疾病完全缓解，行手术治疗后随访35个月未见复发及转移的发生；1例Ⅲb期患者行气管镜局部肿瘤切除术后口服厄洛替尼治疗随访66个月未见进展；另外1例Ⅳ期患者口服厄洛替尼治疗后病情得到控制，随访11个月后病情进展死亡，提示EGFR-TKI对肺MEC有效率较高，但其疗效并不依赖于*EGFR*突变状态。但由于肺MEC的发病率低应用EGFR-TKI的治疗仍属于少数案例，需要进一步的研究证实EGFR-TKI治疗肺粘液表皮样癌的有效性及机制。

既往研究^[1, 5, 12]^表明临床分期、组织学分级、年龄、淋巴结转移情况、肿瘤是否完全切除，术后是否行化疗是影响肺MEC预后的主要因素。本组患者进行单因素生存分析结果显示临床分期、组织学分级、是否行手术治疗是影响预后的因素，年龄、性别、是否吸烟对生存无影响。进一步的多因素分析显示只有临床分期和组织学分级是影响预后的独立危险因素。

综上所述，肺MEC发病率低且缺乏特异性的症状、体征和CT表现，诊断主要依靠术后病理，小标本可结合免疫组织化学结果诊断，*EGFR*基因的突变情况尚需进一步研究。目前肺MEC的治疗方式早期主要依赖手术治疗，晚期病人预后差，尚无好的治疗方式，EGFR-TKI有望成为改善肺MEC预后最具潜力的药物，但尚需要进一步研究。
